# High-throughput sequencing of murine immunoglobulin heavy chain repertoires using single side unique molecular identifiers on an Ion Torrent PGM

**DOI:** 10.18632/oncotarget.25493

**Published:** 2018-07-13

**Authors:** Jean-Philippe Bürckert, William J. Faison, Danielle E. Mustin, Axel R.S.X. Dubois, Regina Sinner, Oliver Hunewald, Anke Wienecke-Baldacchino, Anne Brieger, Claude P. Muller

**Affiliations:** ^1^ Department of Infection and Immunity, Luxembourg Institute of Health, Esch-sur-Alzette, Luxembourg

**Keywords:** high-throughput sequencing, murine IG repertoire, unique molecular barcoding, database benchmarking, IMGT, Immunology

## Abstract

With the advent of high-throughput sequencing (HTS), profiling immunoglobulin (IG) repertoires has become an essential part of immunological research. Advances in sequencing technology enable the IonTorrent Personal Genome Machine (PGM) to cover the full-length of IG mRNA transcripts. Nucleotide insertions and deletions (indels) are the dominant errors of the PGM sequencing platform and can critically influence IG repertoire assessments. Here, we present a PGM-tailored IG repertoire sequencing approach combining error correction through unique molecular identifier (UID) barcoding and indel detection through ImMunoGeneTics (IMGT), the most commonly used sequence alignment database for IG sequences. Using artificially falsified sequences for benchmarking, we found that IMGT's underlying algorithms efficiently detect 98% of the introduced indels. Undetected indels are either located at the end of the sequences or produce masked frameshifts with an insertion and deletion in close proximity. The complementary determining regions 3 (CDR3s) are returned correct for up to 3 insertions or 3 deletions through conservative culling. We further show, that our PGM-tailored unique molecular identifiers result in highly accurate HTS data if combined with the presented processing strategy. In this regard, considering sequences with at least two copies from datasets with UID families of minimum 3 reads result in correct sequences with over 99% confidence. Finally, we show that the protocol can readily be used to generate homogenous datasets for bulk sequencing of murine bone marrow samples. Taken together, this approach will help to establish benchtop-scale sequencing of IG heavy chain transcripts in the field of IG repertoire research.

## INTRODUCTION

The diversity of the immunoglobulin (IG) repertoire is the key feature of the adaptive immune system, enabling it to theoretically combat every possible antigen encountered during an individual's lifetime [1]. With the development of high-throughput sequencing (HTS) it became possible to analyze the IG repertoire at high depth [2–6]. Studies, almost a decade ago, established Roche's 454 sequencer as the first tool of choice for exhaustive characterization of IG repertoires due to its superior read-length [7]. More recently, Illumina's MiSeq and HiSeq sequencers as well as the Ion Torrent Personal Genome Machine (PGM, Thermo Fisher Scientific) provided improved sequencing technologies which can reach across the full V(D)J nucleotide sequence [8]. The different technologies of the sequencers result each in their specific error-rates and -types [7, 9–15]. Illumina's optical sequencing produces mostly nucleotide (nt) transversions and transitions, which can be corrected by building consensus sequences [16]. The 454's pyrosequencing chemistry and the PGMs semiconductor technique mainly introduce homopolymer repeats resulting in insertions and deletions of bases, which can be corrected by gene segment-wise reference alignment [17].

Most sequencing approaches use IG isotype specific constant (C) region primers to translate IG heavy-chain (IGH) (m)RNA into cDNA, which are subsequently amplified using a set of V-region specific primers in a multiplex PCR approach. However, this can result in skewed repertoire assessments due to biased PCR efficacy [8, 14, 18]. In addition, sequencing errors can falsify somatic hypermutation profiles, VDJ germline gene assignment and clonal grouping [8, 19]. Unique molecular identifiers (UID) which tag individual RNA molecules at cDNA transcription level have been used to obtain an unbiased view on the IG repertoire [20–23]. This method also allows thorough error correction by building consensus sequences, albeit at the cost of sequencing depth. In all cases, complex bioinformatic approaches are necessary to perform raw-read processing [24]. Subsequent alignments to germline genes to assign VDJ family genes are in general conducted using the V-QUEST or HighV-QUEST tools available at the ImMunoGeneTics (IMGT) database, which applies an error correction algorithm for insertions and deletions in the process [25, 26].

After the initial proof-of-concept studies, the use of animal models to study the IG repertoire dynamics has been largely ignored [4, 6]. One major factor being the lack of a suitable IGH V-region primer set comparable to BIOMED-2, developed for the human IG repertoire [27]. Yet, animal models offer advantages over human studies, as they are not limited to peripheral blood and have a lower B cell diversity [28–30]. As IMGT provides germline repertoires for various species, we chose to develop a method to profile the IG repertoire of Balb/C mice, one of the most commonly used animal models.

In the present study, the performance of the PGM sequencing platform together with the IMGT HighV-QUEST tool for the assessment of murine IGH repertoires is evaluated. In this context, several novel aspects are examined: first, the IMGT's indel detection and correction algorithm is benchmarked with a set of artificially falsified sequences. Second, a 16-nucleotide single side UID (ssUID) barcoding technique tailored to the PGM sequencing chemistry is introduced together with a swift 1-day library preparation protocol. Third, the PGM's error-rate for sequencing murine IG transcripts with our barcoding strategy and customized data processing is determined.

## RESULTS

### Reference sequences

**Figure 1:**
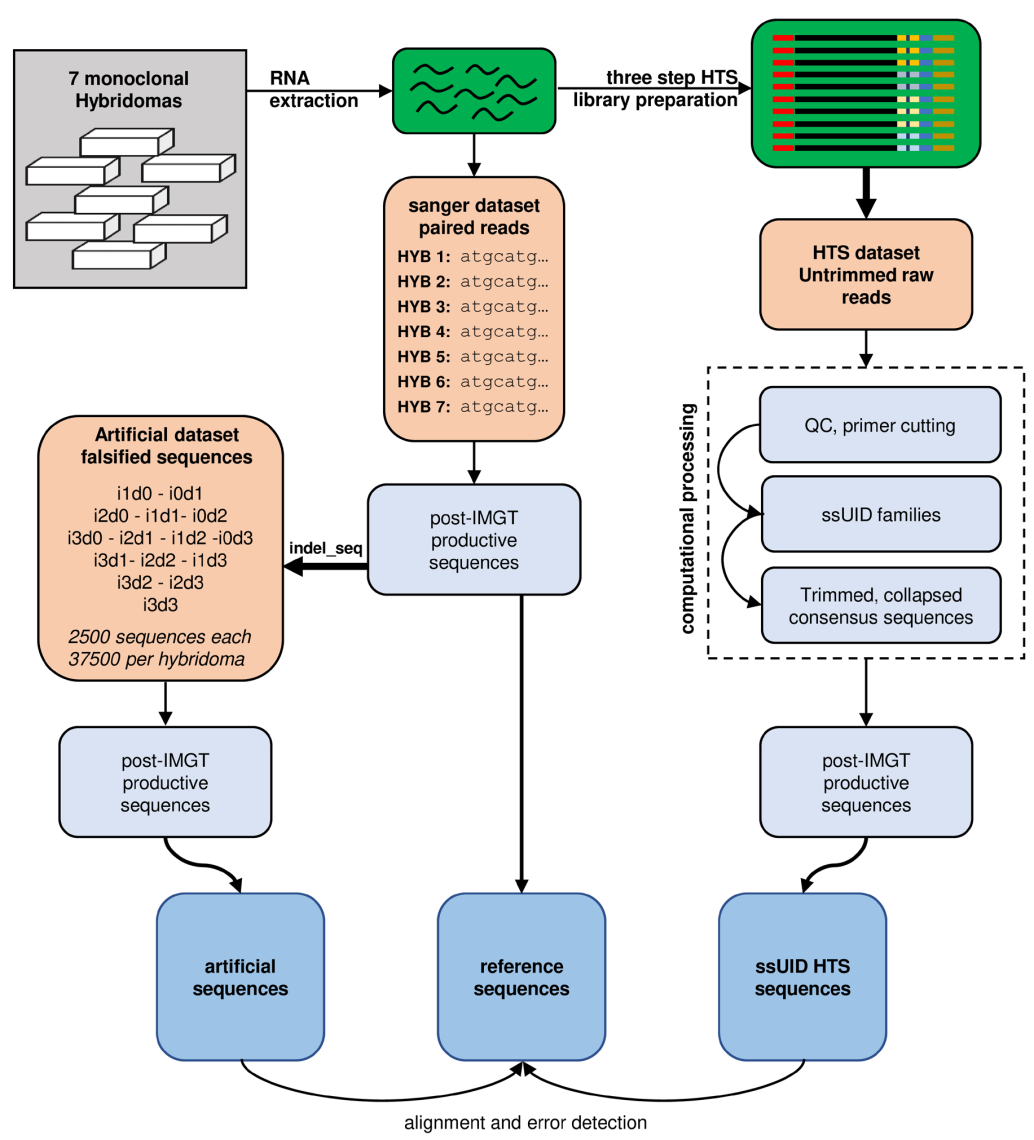
Study design RNA was extracted from 7 monoclonal hybridoma cell lines and reverse transcribed into cDNA. cDNA sequences were determined by Sanger sequencing and submitted to IMGT V-QUEST to determine reference sequences. Reference sequences were artificially falsified using the indel_seq program, introducing up to 3 insertions and 3 deletions. 2500 artificial sequences were generated for each permutation and hybridoma and processed by IMGT HighV-QUEST. Post-IMGT HighV-QUEST sequences were aligned to the references to determine error detection and correction. RNA was also used to generate high-throughput sequencing (HTS) libraries in a three-step library preparation protocol. Single side unique identifiers (ssUID) were introduced during reverse transcription to tag each RNA molecule individually (see also supplementary Figure 3). Libraries were sequenced on an Ion Torrent PGM sequencer with all quality trimming options disabled in the Torrent Suite software. Untrimmed raw sequences were processed with a custom-made bioinformatics pipeline generating consensus sequences per UID family. Collapsed consensus sequences were submitted to IMGT HighV-QUEST and post-IMGT HighV-QUEST sequences aligned to the reference sequences to determine error detection and correction.

A set of 7 monoclonal Balb/C mouse hybridoma cell lines was used to investigate the distribution and influence of insertions and deletions (indels) produced by the IonTorrent PGM sequencing technology on murine IGH repertoire sequencing (Figure 1). Reference sequences were obtained from Sanger sequenced cDNA transcripts of monoclonal hybridoma RNA subsequently annotated, with native germline sequences identified (see Supplementary Table 1) and translated into amino acids by IMGT V-QUEST.

### Distribution of artificial insertions and deletions

**Figure 2:**
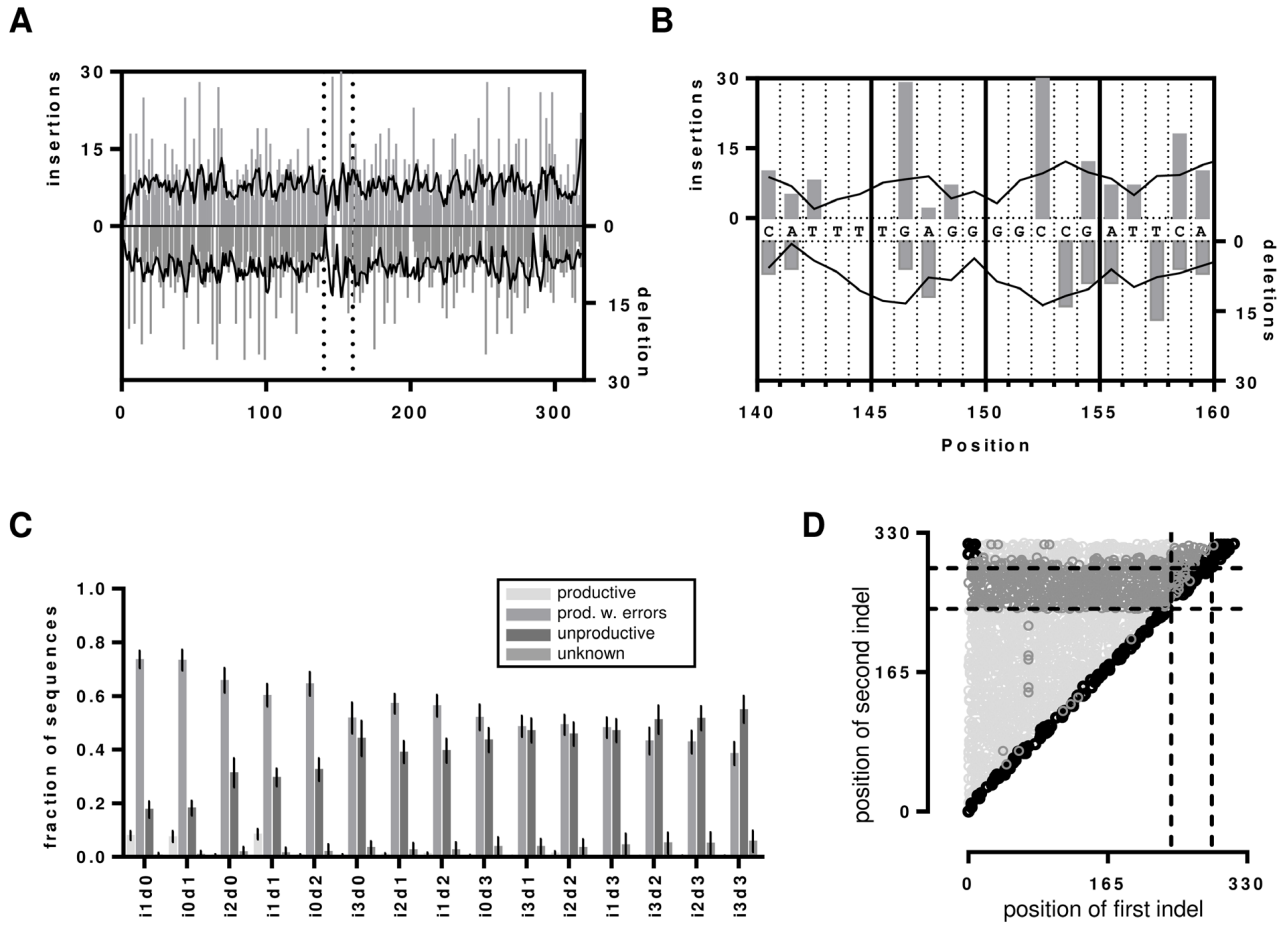
Indels in the artificial dataset **A.** Insertion and deletion events displayed as determined by graphical alignments of the reference sequence to the i1d0 and i0d1 dataset of hybridoma 1. Grey bars represent the actual detected indel and the black line presents the moving average over 4 neighbors. The dotted vertical lines represent the segment that is magnified in **B.** to visualize the problem of determining the position of indels within homopolymer repeats. **C.** Indel detection rates by IMGT HighV-QUEST processing shown as bar chart with error bars indicating the SD over all 7 datasets. **D.** Visualization of frame-shift masking indel proximity in Hybridoma 1 i1d1 dataset. The nt positions of the first and second indel before correction are shown as scatterplot. Dotted lines indicate the position of the IMGT IGH junction. Productive sequences with detected indels are shown in light grey, unproductive sequences are shown in dark grey. Sequences without detected errors are shown in black. The remaining i1d1 indel proximity graphs are shown in the supplementary Figure 1.

To investigate the influence of indels on IMGT HighV-QUEST processing of an IGH sequence, we generated a benchmark dataset from the reference sequences that contained artificially introduced indels at random positions (Supplementary Table 2). To cover each position within a 300 nt sequence with minimum 90% certainty, at least 2398 erroneous variants are required [31]. Therefore, we generated 2500 artificial, randomly flawed sequences for each permutation of 0-3 insertions and/or deletions (indels, annotated as i1d0, i0d1, i1d1 … i3d3), resulting in a total of 37500 artificial sequences per original hybridoma sequence with indels ranging from 1 to 6 events. Indels were homogenously present as determined by graphical reference alignment (Figure 2A). Uncovered positions resulted from indels within homopolymer stretches which were always assigned to the beginning of such a nucleotide repeat region (Figure 2B).

### IMGT HighV-QUEST VDJ nucleotide error detection

**Figure 3:**
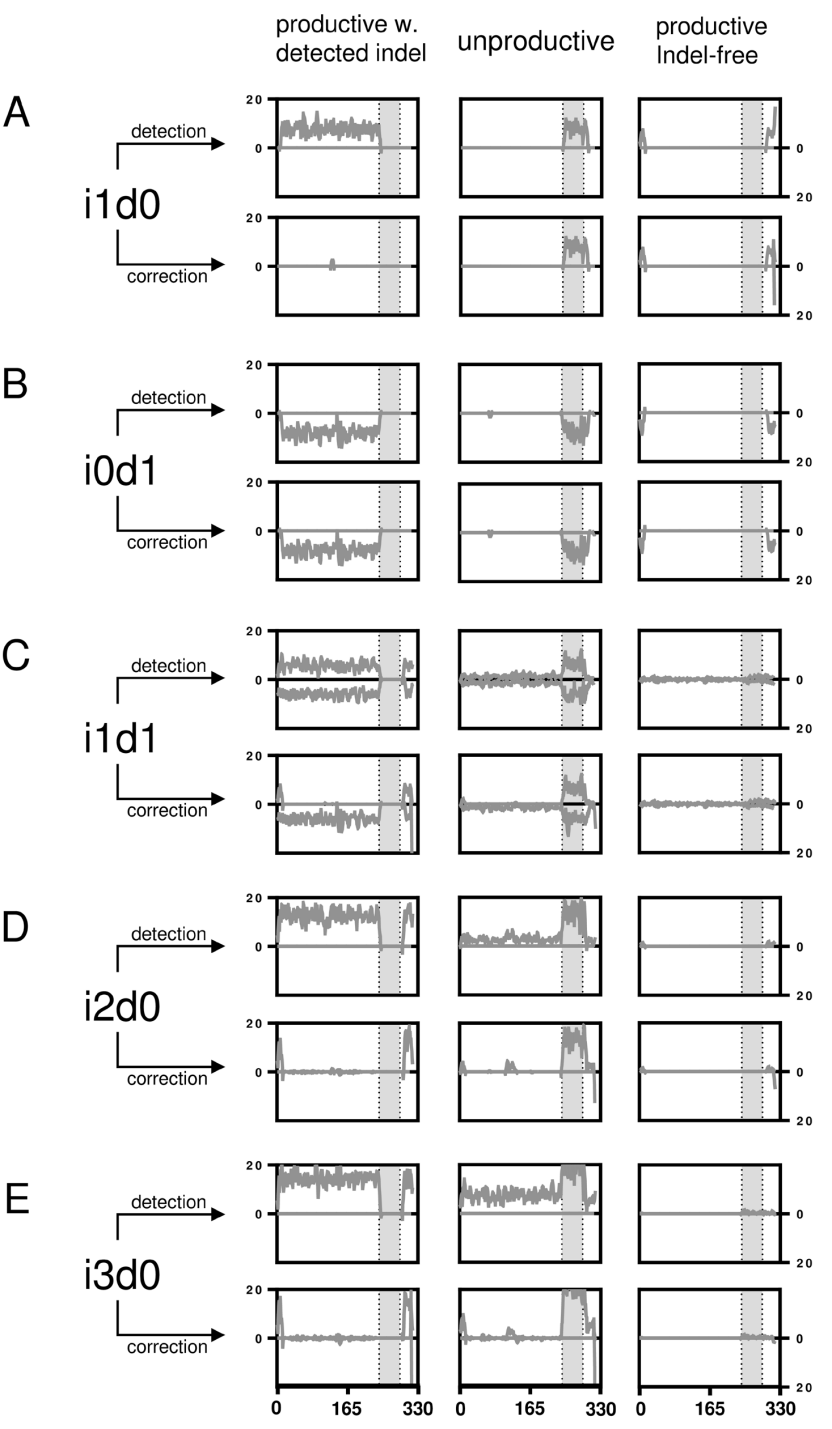
Artificial indel set alignments Number of indels are shown per nucleotide position before and after IMGT HighV-QUEST error correction for artificially falsified Hybridoma 1 sequences separated by productivity as returned by IMGT HighV-QUEST. **A.** The number of indels for the i1d0 dataset are shown per nucleotide position as line plot (smoothened over 4 neighbors). The grey area marks the IGH VDJ junction. **B.**-**E.** like (A) but with different permutations as indicated. The remaining permutations are displayed in the supplementary Figure 2.

As each sequence of the benchmark system contained indel errors, all sequences marked by IMGT HighV-QUEST as productive were falsely categorized as error free. In general, IMGT HighV-QUEST correctly recognized 97.9% (± 2.9%) of the introduced indels over all datasets and categorized the sequences then either as productive with detected indels, unproductive or unknown (Figure 2C). Interestingly, only the sets with one insertion and/or deletion (i1d0, i0d1 and i1d1) exhibited elevated numbers of unrecognized indels. For these IMGT HighV-QUEST falsely returned 8% (±1.8%) of the sequences as productive, whereas for all other datasets it was only 0.7% (± 0.4%). Such undetected indels were found at the beginning and the end of the sequence or across the whole sequence for i1d1 datasets due to indels in close proximity to each other masking the frame-shifts (Figure 2D, Figure 3, supplementary Figure 1 and 2). The number of unproductive sequences increased with the number of indel events, regardless of their composition. Accordingly, the number of productive sequences with detected indels decreased. Less than 50% of sequences with more than 3 indels, were retained. Indels were homogenously distributed in the uncorrected productive sequences with detected errors until about 4/5^th^ of the sequence lengths while the opposite is true for the uncorrected unproductive sequences (Figure 2D, Figure 3 and supplementary Figure 2). This section of the sequence coincides with the IMGT IGH junction which encodes for the CDR3 [32]. Accordingly, upon detecting an indel in the IGH junction, IMGT HighV-QUEST categorized the sequence as unproductive and no corrective attempts were made.

### IMGT HighV-QUEST VDJ nucleotide error correction

**Figure 4:**
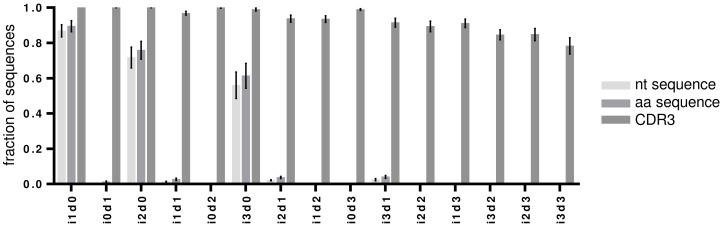
Correction of artificially introduced indels by IMGT HighV-QUEST The fraction of correct sequences after IMGT HighV-QUEST processing for each artificial indel permutation are shown as bar charts for nucleotide (nt), amino acid (aa) and CDR3 amino acid sequences. Error bars indicate SD over all 7 artificial datasets.

Upon detection of an indel, IMGT HighV-QUEST tries to correct it by alignment to its closest germline. The efficacy of this process was investigated by aligning the sequences with detected indels to determine the number of correctly resolved sequences (Figure 3, Figure 4 and supplementary Figure 2). A thorough error reduction was observed for up to three insertion errors in datasets without deletions, returning 87% ± 3.2% (i1d0), 72% ± 5.5% (i2d0) and 56% ± 7.0% (i3d0) of productive sequences as correct (Figure 4). Within these sequences indels that were not corrected by the IMGT HighV-QUEST were mainly found at the beginning and end of the sequence (Figure 3A, 3D, 3E). In the case of deletions, the IMGT HighV-QUEST correction introduced a gap for the missing nucleotide as the original nucleotide was unknown. Consequently, the number of correct sequences found in datasets with mixed insertions and deletions is very low (i1d1: 1% ± 0.3%, i2d1: 2% ± 0.3%, i3d1: 2% ± 0.6%, i2d2 and i3d2 < 1%). Nevertheless, in these datasets, the insertions within the sequences were always reduced (Figure 3C and supplementary Figure 2). No correct sequence could be identified in deletion-only datasets (Figure 4).

### IMGT HighV-QUEST VDJ amino acid error correction

Theoretically, translated amino acids are less influenced by sequencing errors because of the redundancy of the genetic code. Thus, most amino acid translations were returned correctly in the case of insertion-only datasets and with slightly higher numbers compared to the nucleotide datasets (mean correct amino acid sequences for i1d0: 89% ± 2.9%, i2d0: 76% ± 4.7%, i3d0: 61% ± 6.5%, Figure 4). Higher numbers of correct translations were observed in mixed indel datasets than for the corresponding nucleotide datasets (i1d1: 3% ± 0.7%, i2d1: 4% ± 0.6%, i3d1: 4% ± 0.8%, i2d2 and i3d2 < 1%, Figure 4). Interestingly, some amino acid translations were found to be correct for the i0d1 datasets (1% ± 0.5%, Figure 4). Deletion-affected datasets were usually returned with the wrong amino acid sequence by the underlying algorithm. During IMGT HighV-QUEST processing, nucleotide deletions rendered the whole codon triplet elusive and were translated as gaps in the amino acid sequence.

Remarkably, the CDR3 proved to be protected chiefly from insertions and deletions through a more conservative correction approach of the IMGT HighV-QUEST algorithm for this part of the sequence. As mentioned above, detected indels within the IGH junction, and thus the CDR3, corrupted the entire sequence as unproductive (Figure 3 and supplementary Figure 2). Culling attempts by IMGT HighV-QUEST turned out to be largely successful (100% correct CDR3s for up to 3 insertions or 3 deletions). Even for the i3d3 indel permutation, IMGT HighV-QUEST returned 78% ± 4.3% correct CDR3s (Figure 4), by removing all those sequences where indels were detected in the CDR3 encoding nucleotides. Datasets with simultaneous insertions and deletions showed in general lower numbers of correct CDR3 sequences (range 78-97%). This resulted from sequences where indels were introduced in close proximity of each other, producing no detectable frameshift within the IGH junction (Figure 2D). While invisible for the IMGT HighV-QUEST algorithm, they were observed as variants of the correct CDR3 amino acid sequence.

Taken together the above data show, that IMGT HighV-QUEST processing exhibits adequate detection of indels through frame-shifts in mouse IGH nt sequences. Consequently, frame-shift masking error compositions cannot be detected and result in amino acid changes in the translations. IMGT's HighV-QUEST indel correction proved to be reliable for single insertions. However, the impossibility to correct for deletions and larger indel permutations makes consideration of sequences categorized as “productive with detected indels” unfavorable.

### HTS of hybridoma ssUID libraries

**Figure 5:**
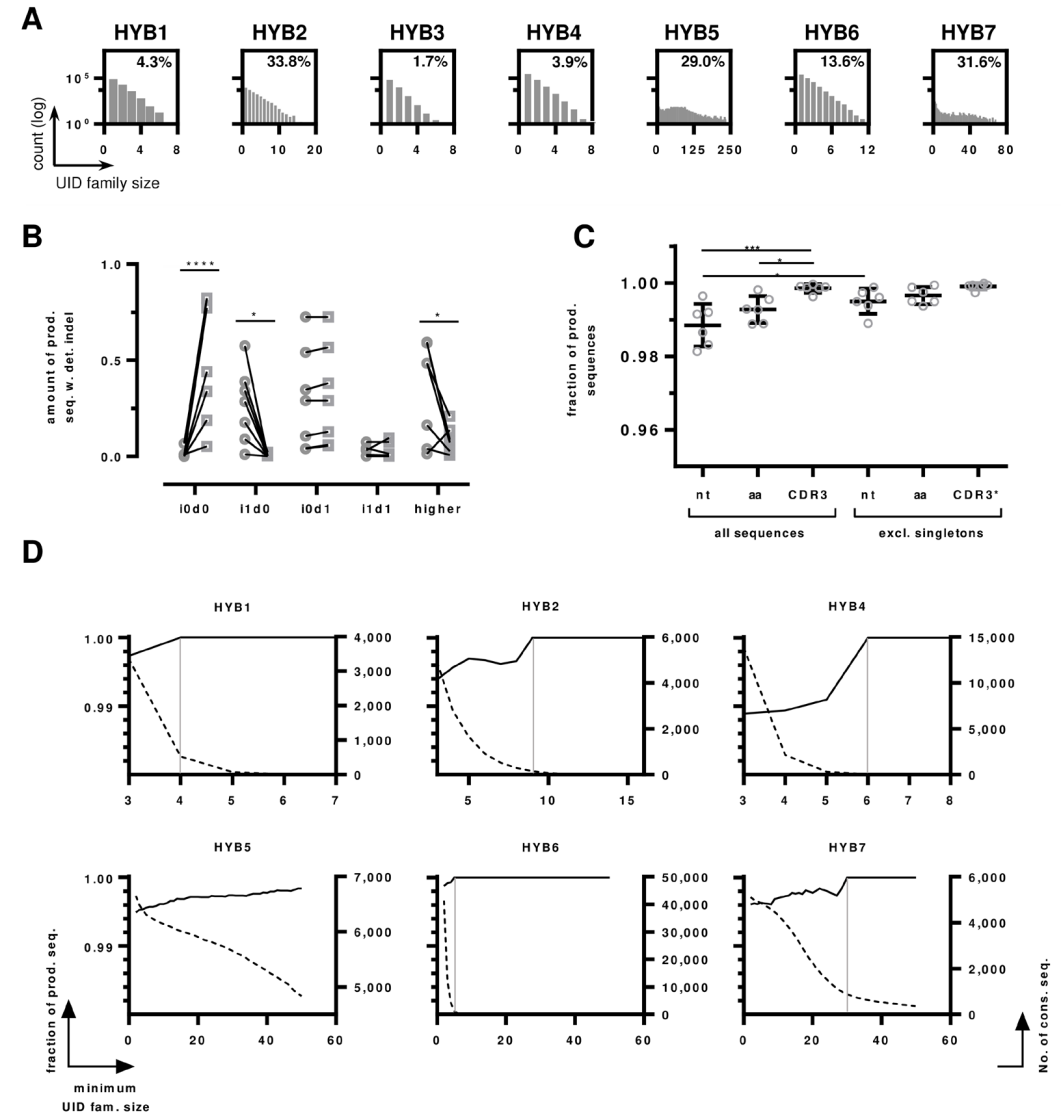
HTS data of monoclonal hybridomas libraries **A.** UID family size distributions per sample. The number of UID families (log transformed) is plotted by the number of reads assigned to a ssUID per hybridoma. The amount of UID families containing a minimum of 3 reads are indicated as percentage value. **B.** Indel distributions on productive sequences with detected errors before and after IMGT HighV-QUEST processing. The amount of indel-free (i0d0), single insertions (i1d0), single deletions (i0d1), one single insertion and deletion (i1d1) and higher permutations (“higher”) are shown as fraction of productive reads with detected indels before (circles) and after (squares) IMGT HighV-QUEST error correction. Statistical differences are indicated with **** *p* < 0.0001, * *p* < 0.05 determined by multiple two-tailed *t*-test with Holm-Sidak's method to account for multiple testing. **C.** The influence of removing singletons on the number of error-free sequences in the productive dataset. The fractions of total sequences without detected indels are shown as boxplot with mean and ± SD. Data are shown for all nucleotide sequences (nt), amino acid sequences (aa) and CDR3s for all sequences and data without singleton sequences. CDR3* indicates that for this set, singletons were determined on the full-length amino acid sequences. P values are indicated *** *p* < 0.001, * < 0.05, One-way ANOVA with Sidak's post-hoc test. All other differences were not statistically significant. **D.** Influence of UID family size on the number of correct sequences. The number of correct sequences are shown as black line per minimum UID family size (left y-axis). The number of consensus sequences are shown as dotted line per minimum UID family size (right y-axis). The UID family size at which all sequences are correct is indicated by a grey vertical line for Hybridoma 1,2,4,6 and 7, the dataset of Hybridoma 5 does not reach 100% correct sequences.

Next, the IMGT HighV-QUEST tool and a PGM-tailored data processing pipeline developed by our group were tested using real HTS datasets derived from 7 monoclonal hybridomas (Figure 1). The HTS libraries were prepared using an IonTorrent PGM tailored single-side UID approach (supplementary Figure 3) allowing for error correction through building consensus sequences from all reads within a UID family [33, 34]. The ssUID barcodes, together with the C-region primer and appropriate ‘GATC’ spacer, were correctly identified at the sequencing start site of 99.12% ± 0.56% of the usable reads containing a sample specific MID (Table 1). Between 146,010 and 739,854 reads were obtained per sample, with varying ssUID family size distributions (Figure 5A). After raw data processing, 1,431 to 47,169 consensus sequences were retained per hybridoma (Table 1) and uploaded to IMGT HighV-QUEST.

**Table 1:** HTS hybridoma datasets pre-IMGT

Set	Chip	reads with MID	reads with primer & UID	consensus sequences
**HYB1**	A	207,753	206,929	4,159
**HYB2**	A	147,634	146,010	7,760
**HYB3**	A	222,929	222,100	1,431
**HYB4**	A	882,242	877,823	16,643
**HYB5**	B	747,827	733,258	7,319
**HYB6**	B	743,465	739,854	47,169
**HYB7**	B	204,348	201,619	5,426
**BM1**	C	679,600	581,983	37,877
**BM2**	C	592,044	533,839	37,388
**BM3**	C	566,441	517,149	32,748
**BM4**	C	722,267	643,847	38,635

### IMGT HighV-QUEST processing of HTS hybridoma datasets

The majority of the sequences returned by IMGT HighV-QUEST were categorized as productive (75.8% ± 22.6%) and 10.9% (± 9.6%) were categorized as productive with detected indels (Table 2). The remaining sequences were either categorized as unproductive or unknown/else. To investigate the undetected or uncorrected errors within the two productive categories, sequences were aligned to their corresponding references. For Hybridoma 3, which had the poorest UID distribution (Figure 5A), only 26.8% of the sequences were classified as productive and 68.8% unproductive (Table 2). This hybridoma was therefore excluded from further analysis.

**Table 2:** HTS hybridoma hybridoma datasets classifications by IMGT HighV-QUEST

Set	prod. seq.	%	prod. w. det. indel	%	unprod	%	unknown/ else	%
**HYB1**	3,328	79.6%	622	14.9%	127	3.0%	102	2.4%
**HYB2**	4,866	62.7%	2,449	31.6%	250	3.2%	195	2.5%
**HYB3**	381	26.6%	62	4.3%	984	68.8%	4	0.3%
**HYB4**	13,515	81.2%	2,215	13.3%	329	2.0%	584	3.5%
**HYB5**	6,697	91.5%	281	3.8%	51	0.7%	290	4.0%
**HYB6**	43,767	92.8%	3,009	6.4%	287	0.6%	106	0.2%
**HYB7**	5,216	96.1%	111	2.0%	15	0.3%	84	1.5%
**Mean**	11,110	75.8%	1,250	10.9%	292	11.2%	195	2.1%
**SD**	13,842	22.6%	1,165	9.6%	303	23.5%	180	1.4%

In the group of productive sequences with detected errors, the IMGT HighV-QUEST indel correction algorithm improved the number of correct sequences by 54.1% to on average 55.3% (± 32.0%, Figure 5B). As expected, IMGT HighV-QUEST corrected most sequences that contained single insertions efficiently, reducing these errors from average 25.2% (± 24.3%) to 0.48% (± 0.72%, *p*-value = 0.0027, two-tailed *t*-test in Graphpad Prism, using Holm-Sidak's method [35] to account for multiple testing with alpha = 5%, Figure 5B). Single deletions were found at somewhat higher rates than single insertions (29.9% ± 24.3%) of the sequences. They increased slightly after IMGT HighV-QUEST error correction (31.6% ± 24.1%), as insertions of higher indel permutations were corrected, leaving only deletions in the sequences. Accordingly, these higher permutations were found in 33.8% (± 23.8%) of the sequences before error-correction and reduced to 8.8% (± 6.3%) afterwards. While the detection of indel errors in the sequences by IMGT HighV-QUEST was efficient, the remaining errors after correction still affected 44.7% ± 32.2% of the sequences. As described for the benchmarking sequences above, this makes further consideration of sequences marked as “productive with detected indels” inadvisable.

Sequences marked as productive without detected indels are not modified by IMGT HighV-QUEST but can nonetheless contain indel and nucleotide substitution errors. IMGT HighV-QUEST does not detect ambiguous nucleotides as errors but marks them as silent mutations. On average 2.2% (± 1.6%) of the consensus sequences in the productive dataset without detected indels contained ambiguous nucleotides (Table 3), which were discarded from the datasets. Most of the remaining sequences were indeed error-free (98.8% ± 0.5%, Figure 5C). The other 1.2% contained on average 0.2% (± 0.1%) i1d1 indels in close proximity to each other, masking frameshifts. Some sequences showed single insertions (0.1% ±0.2%) and deletions (0.15% ± 0.13%), either at the beginning or the end, without detectable frameshift. The remaining false sequences contained nucleotide substitutions, with the majority being transversions (0.5% ± 0.3%) and very few transitions ( < 0.1%). As described by Shugay and coworkers, such substitutions originate from dominating polymerase errors occurring early during the amplification [33]. As polymerase errors are occurring at relatively random positions, it is stochastically unlikely, that the same errors are found repeatedly within a dataset and can thus be accounted for by considering only consensus sequences that appear more than once in the final dataset [33, 34]. Following this approach, the data was reassessed, excluding singleton consensus sequences. This reduced the number of total sequences in the datasets by 0.8% (± 0.4%). The number of transversions was reduced significantly by 0.3% to 0.16% (± 0.19%, *p*-value = 0.008, two-tailed *t*-test in Graphpad Prism, using Holm-Sidak's method to account for multiple testing with alpha = 5%, data not shown). Consequently, the number of error-free sequences improved significantly by 0.7% to 99.5% (± 0.3%, *p*-value < 0.0001, two-tailed *t*-test, using Holm-Sidak's method to account for multiple testing with alpha = 5%).

**Table 3:** Ambiguous nt in HTS hybridoma datasets

	HYB1	HYB2	HYB4	HYB5	HYB6	HYB7	Mean	SD
**Amb nt**	26	135	97	90	2289	148	464	817
**%**	0.8	2.6	0.7	1.3	5.2	2.8	2.2	1.6

The number of reads per UID, referred to as UID family size, is crucial to obtain reliable consensus sequences [34]. Increasing the minimum number of required reads per UID family improved the amount of correct sequences, reaching 100% for all hybridomas, except Hybridoma 5, albeit with different UID family sizes (Figure 5D). However, with increasing minimum UID family sizes, the number of sequences decreased exponentially. Consequently, at the point of reaching 100% correct sequences, on average only 7.9% (± 7.1%, excl. Hybridoma 5) of the sequences remained (Figure 5D). According to our data, keeping a minimum UID family size of 3 provided adequate accuracy and throughput when using an IonTorrent PGM. As expected, the number of correct amino acid sequences was higher (99.3% ± 0.3%) than the amount of correct nucleotide sequences (Figure 5C). An average of 0.6% (± 0.4%) of the sequences was subject to amino acid changes. Excluding singleton amino acid sequences increased the number of correct amino acid sequences to 99.7% (± 0.2%), but this increase was not statistically significant. CDR3 amino acid sequences were returned almost entirely correct (99.85% ± 0.11%, Figure 3C), increasing to 99.91% (± 0.08%) when singleton full-length amino acid sequences were excluded.

### HTS and processing of bulk mouse BM samples

**Figure 6:**
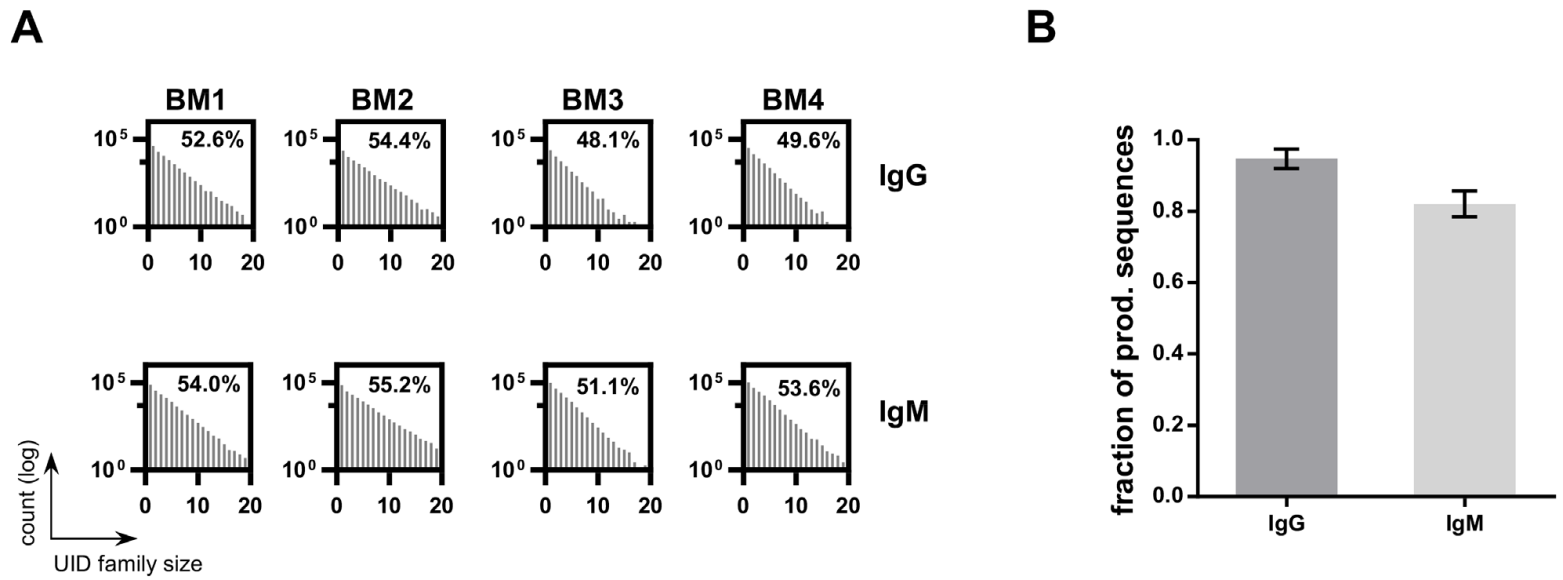
HTS data on bulk BM libraries **A.** UID family size distributions per sample. The number of UID families (log transformed) is plotted by the number of reads assigned to a ssUID per bulk BM library. The amount of UID families containing a minimum of 3 reads are indicated as percentage value. **B.** The number of sequences retained after excluding singleton for bulk BM HTS datasets. Bars represent the fraction of total sequences after excluding singletons for IgG (grey) and IgM (light grey), respectively. Error bars indicate the SD over all four datasets.

To verify our ssUID approach for bulk sequencing, mouse bone marrow samples from four mice were processed with C -region specific primers targeting mouse IgG and IgM isotype BCRs (Supplementary Table 3). The ssUID barcodes, together with C-region primers and ‘GATC’ spacer, were identified at the sequencing start site of 89.1% ± 2.1% of the usable reads containing a sample specific MID (Table 1). All samples had homogeneous ssUID family size distributions for both isotypes (Figure 6A). On average, 51.2% (± 2.5%) and 53.5% (± 1.5%) of the ssUID families for IgG and IgM, respectively, contained more than two sequences and were considered for consensus building. This resulted in 5,170 to 12,025 consensus sequences were retained for IgG samples and 25,825 to 31,612 consensus sequences for the IgM isotype per isotype after raw data processing (Table 4) which were subsequently uploaded to IMGT HighV-QUEST.

**Table 4:** HTS BM datasets classifications by IMGT HighV-QUEST

Set	prod. seq.	%	prod. w. det. indel	%	unprod	%	unknown/ else	%
**BM1**	30,003	79.2%	5,585	14.7%	1,015	2.7%	1,274	3.4%
**BM2**	30,971	82.8%	5,214	13.9%	999	2.7%	204	0.5%
**BM3**	27,990	85.5%	3,777	11.5%	776	2.4%	205	0.6%
**BM4**	28,371	73.4%	8,747	22.6%	1,241	3.2%	276	0.7%
**Mean**	29,335	80,2%	5,832	15,7%	490	1.3%	1,008	2.7%
**SD**	1,210	4.5%	1,814	4,2%	454	1.2%	165	0.3%

IMGT HighV-QUEST returned 80.2% (± 4.5%) of the sequences as productive and 15.7% (± 4.2%) of the sequences contained indels, as detected by the IMGT HighV-QUEST indel identification algorithm (Table 4). On average, 2.7% (± 0.3%) of the sequences were found unproductive and the remaining were categorized as unknown/else. After IMGT HighV-QUEST processing, most of the IgG sequences were found with two or more copies (94.7% ± 2.4%) and as expected, less for the IgM sequences (82.0% ±3.1%) (Figure 6B). As determined above, these singlet sequences should be removed from final data to obtain highly reliable sequencing results.

## DISCUSSION

Investigation of IG repertoires by HTS is challenging both with respect to the library preparation as well as sequencing error assessment and data processing. Using artificially falsified sequences, we show here that the IMGT HighV-QUEST indel detection algorithm is efficient while the IMGT HighV-QUEST indel correction algorithm only corrects single insertions sufficiently. We confirm the utility of the IonTorrent PGM to assess murine IGH repertoires with high confidence, using a dedicated library preparation protocol with a PGM-tailored 16 nt single side unique identifier (ssUID) barcoding technique. Our data show that appropriate data processing reduced the error rate of PGM-sequenced IGH repertoires to less than 0.5% false nucleotide and amino acid sequences, and to less than 0.01% false CDR3 sequences per dataset.

Sequencing of IGH repertoires requires a thorough assessment and correction of platform inherent sequencing errors [7, 9, 12–15]. Using the IMGT HighV-QUEST tool for reference alignment, the indel errors of the utilized Ion Torrent PGM sequencing platform can theoretically be detected through the resulting codon frame-shifts [17]. The VDJ structure of the IGH sequence facilitates indel detection by frame-shift, since gene segments can be aligned separately. In our study, the IMGT HighV-QUEST algorithm successfully detects 97.9% of all indels, regardless of their composition, only single insertions or deletions at the beginning or the end of the sequences (7.9% and 7.5%, respectively), or i1d1 compositions in close proximity to each other could not be identified (8.5%). IMGT HighV-QUEST tries to correct detected insertions subsequently by removing the false nucleotide(s) according to the predicted germline sequence. In the artificially falsified datasets of our study insertion-only errors were corrected by the IMGT HighV-QUEST algorithm with 87% (i1d0), 72% (i2d0) and 56% (i3d0) efficiency. Deletions, on the other hand, are more difficult to recover since the missing nucleotide cannot necessarily be inferred from the germline sequence with sufficient confidence. Consequently, artificially introduced deletions were not corrected by IMGT HighV-QUEST. Also, for sequences with mixed insertions and deletions only the nucleotide insertions were corrected by IMGT HighV-QUEST leaving the sequence erroneous. Furthermore, indels could impair the alignment process by changing the identification of the closest germline. While we observed more potential germlines to be suggested by IMGT HighV-QUEST for the higher indel permutations, the selected assignment by IMGT HighV-QUEST of the closest germline did not change even with the highest permutation of indels (i3d3) tested (data not shown). Taken together, these data indicate that detection of indels by IMGT HighV-QUEST is highly efficient and sequences categorized as “productive” without detected errors are almost entirely indel-free. The low efficiency of the indel correction algorithm makes it inadvisable to take productive sequences with detected indels into account for any downstream analysis. These correspond to about 10% of the final HTS consensus sequences in our study.

HTS library preparation using multiple primers during template amplification can significantly bias the repertoire composition [14, 19]. This bias is essentially removed by UID barcoding, but the approach reduces sequencing depth at the same time [34, 36–38]. In our study, the raw sequencing depth does not influence the relative number of correct sequences while the average UID family size proved to be crucial. For instance, Hybridoma 3, although having only the 4^th^ lowest amount of raw-reads, lacked eligible UID family sizes ( > 2 sequences per UID). For Hybridoma 3, less than 1.7% of the UID families had more than 2 members, resulting in the poorest error correction rate during sample processing, potentially because of low amounts of IGH encoding mRNA molecules. While the UID family sizes for the presented Hybridoma sequencing datasets vary largely, the bulk sequencing experiment generated homogeneous datasets with around 50% eligible reads. We were unable to determine the original cause of the poor performance of Hybridoma 3 for HTS which demonstrates, that it is crucial to critically follow samples throughout the entire raw data processing. As for this Hybridoma 3, it became evident that the dataset had low quality only after IMGT HighV-QUEST processing, returning just 26.6% of the consensus sequences as productive. Datasets of higher complexity than single sequence Hybridoma libraries could be even more elusive. We thus conclude from our data, that for applying a UID family-wise consensus building approach, samples with low numbers of eligible consensus reads after pre-IMGT HighV-QUEST processing or unusually high numbers of unproductive sequences ( > number of productive sequences) post-IMGT HighV-QUEST alignment should be discarded from further analysis.

For grouping reads by UID families, it is essential to identify the UID tags correctly [34, 38]. The PGM sequencing chemistry is unidirectional, starting with the sequencing adapter A. Comparable protocols for the Illumina sequencing platforms usually consist of UID tags at the beginning and the end of the amplicon sequence [39]. We chose to introduce the 16 random nucleotides of the UID tag at the sequencing start site as the PGM semiconductor technology is significantly less accurate towards the end of the sequence [40]. We included a 4-nucleotide spacer as junction into the UID tag resulting in the N_8_-GATC-N_8_ ssUID layout of this study. Like this, we address that the PGM indel rate increases in homopolymer stretches with their length [41], in particular when homopolymers are longer than 8nt [42]. While breaking potential homopolymer patterns within the UID, this design also reduces the number of mistakes during primer synthesis and allows to generate sets of primers with individual spacers that could be used to tag different experiments.

Nucleotide substitution errors are the most difficult to account for in HTS IG repertoire approaches and can critically falsify somatic hypermutation profiles [16, 24]. They can originate from mixed events of adjacent insertions and deletions, which cannot be detected by the IMGT HighV-QUEST algorithm or are introduced as mistakes by the sequencing platform. UID barcoded RNA transcripts allow us to address this problem [8, 33, 34, 39]. B cells contain up to several thousands of identical IG RNA molecules that are each individually tagged by a UID [39, 43]. Therefore, a HTS run provides a snapshot of the relative abundance of RNA transcripts [16]. Comparable to procedures used for identification of single nucleotide polymorphisms (SNP), single occurrences of nucleotide substitutions can be ruled out as artifacts and only transcripts above a certain copy threshold should be retained [43]. Our data show that considering sequences with at least 2 copies in the final dataset improves the proportion of correct sequences by 0.7% to 99.5% by reducing the number of sequences by merely 0.8%. Compared to Hybridoma HTS datasets, more sequences are removed by this step from bulk BM IgG and IgM data, with IgM isotype exhibiting the strongest reduction of sequences (18%). This is expected by the higher diversity of these samples, with IgM isotype BCR carrying B cells being mainly naïve cells with little clonal expansion. However, as the sequences in the datasets from monoclonal Hybridomas are all derived from identical RNA molecules, it makes it stochastically more likely, that the same indel error appears several times. Thus, it can be expected that excluding singletons would increase the number of correct sequences in the bulk B cell derived datasets even more, where less sequences are derived from identical RNA molecules.

For large scale HTS experiments spanning several treatment groups, it is essential to have a reliable library preparation protocol resulting in sequencing libraries with similar depth and limited variation to reduce potential batch effects. The presented workflow returned very homogeneous datasets for libraries prepared from bulk murine BM samples. Even with both isotype primers applied together during reverse transcription, the BM samples showed comparable numbers of reads and sequences throughout the entire data processing approach with little variation. Approximately half of the sequences are lost because they belong to ssUID-families with only a single member. As determined through HTS of the monoclonal hybridoma libraries, those sequences are not reliable and thus should be excluded from further processing. We tried to increase the number of eligible ssUID families by increasing the number of PCR cycles in the amplification step but found that it reduced overall sequencing depth as average family size increased drastically (data not shown). Overall, the presented workflow generates robust and homogeneous data for bulk sequencing approaches of murine BM samples.

In conclusion, we have demonstrated that using our ssUID library preparation in combination with the IMGT database, the PGM sequencing platform can be efficiently used to assess murine IGH repertoires. Considering only consensus sequences with at least two copies in the final dataset improved the sequence quality considerably. Taken together, this approach allowed to obtain highly reliable IGH sequences, with more than 99% confidence in general and 99.9% confidence for the correct CDR3 sequences. The protocol and sample processing strategies described in this study will help to establish the benchtop-scale Ion Torrent sequencing technology of animal models in the field of immunoglobulin repertoire research.

## MATERIALS AND METHODS

### Animals

All animal procedures were in compliance with the rules described in the Guide for the Care and Use of Laboratory Animals and accepted by the ‘Comité National d’Éthique de Recherche’ (CNER, Luxembourg). Balb/c mice (10-week old, female) were obtained from Harlan (Horst, NL) and acclimatized for 1 week. Animals were kept under timed 12h light/dark cycles at 22 °C and 40% relative humidity with food and water available *ad libitum*.

### RNA extraction

RNA was extracted with Trizol LS/chloroform (Thermo Fisher Scientific, Waltham, USA) method from seven monoclonal hybridoma cell lines (produced from Balb/C mice in house) with 10^6^ cells each. DNA was digested using the DNAfree kit (Thermo Fisher Scientific), RNA was further purified using Agencourt^®^ RNAclean XP beads (Analis, Suarlée, BE) and quantified on a NanoDrop^®^ Spectrophotometer (ND1000, Isogen Life Science, De Meern, NL). RNA was either directly used for library preparation or stored at -80°C. For bulk bone marrow samples, lymphocytes were first isolated by density-gradient centrifugation (ficoll^®^ Paque Plus, Sigma-Aldrich) from bone marrow washes. Samples were then processed the same way as for the hybridomas.

### Reference sequences

Hybridoma cDNA transcripts were obtained using mouse constant region IgG primer (Supplementary Table 3) in a Superscript III (Thermo Fisher Scientific) reverse transcription following the manufacturer's instructions for templates with high GC content. Transcripts were Sanger-sequenced (3100 Avant, Thermo Fisher Scientific) using constant region IgG and V-region primers (Supplementary Table 3). Forward and reverse sequences were aligned and submitted to IMGT V-QUEST (http://www.imgt.org, [44]) to verify the nucleotide sequence and to translate into amino acids. These sequences were subsequently used as reference sequences in alignments and artificial error insertion experiments.

### Datasets with artificial insertions and deletions

Artificial datasets were generated using the Biopieces indel_seq package (http://www.biopieces.org). For each of the original 7 hybridoma sequences, 2500 error-containing sequences were generated by combining 0-3 insertions and 0-3 deletions, obtaining a total of 37500 artificial sequences per hybridoma. For every set, indel-type and -position were determined by alignment to the original sequence to ensure homogenous error distributions. All artificial datasets were uploaded to IMGT HighV-QUEST and sorted by annotation: IMGT HighV-QUEST annotates correct sequences as productive. Sequences with a detected indel (frameshift, stop codon) are marked as “productive (see comment)” if the error can be corrected (referred to as “productive with detected errors”). Sequences with uncorrectable errors are classified as “unproductive”. If no fitting germline can be found sequences are marked as “unknown” or “no result” (referred to here as “unknown/else”). The remaining indels on nucleotide level and amino acid changes were determined using the SeqAn library [45] in a custom-made C++ reference alignment program. For datasets with one insertion and one deletion (i1d1) the positions of the indels were determined by position-wise mismatch detection using a custom made Biopython [46] script. Upon detection, the nucleotide positions were returned, and the process repeated with reverse complement sequences.

### Library preparation and HTS

Approximately 100ng (as determined by Nanodrop^®^) of total RNA per hybridoma or bone marrow was used for library preparation. We adapted the UID labeling method developed by Vollmers et al [39] to our PGM sequencing system (supplementary Figure 3). RNA was reverse transcribed using Superscript III reverse transcriptase, according to the manufacturer's instructions, using multiplex identifiers (MID) and UID tagged mouse constant region (IGHγ) primers elongated by partial PGM sequencing adapter pA (Supplementary Table 3). The MID tag allowed multiplexing of several samples on one sequencing chip. The UID tag consists of two times 8 random nucleotides separated by a “GATC” spacer (N_8_-GATC-N_8_). With this UID tag each RNA molecule targeted by the primer is uniquely labeled (see [33, 39] for detailed theoretical descriptions). The RT reaction mixtures were split into two equal second strand synthesis reactions using Phusion^®^ High-Fidelity DNA polymerase (NEB, Massachusetts, USA) with a mouse IGH V-region primer mix (Supplementary Table 3). The reaction conditions were as follows: 98°C 2min, 50°C 2min, 72°C 10 min in a single cycle reaction. Both reaction aliquots were combined and purified twice using Agencourt^®^ AMPure^®^ XP beads (Analis) in a 1:1 (v/v) ratio to remove primer traces. Libraries were subsequently amplified with a Q5^®^ Hot Start High-Fidelity DNA polymerase (NEB) using the full-length Ion Torrent PGM sequencing adapters A and P1 as primers (Supplementary Table 3) with the following conditions: 98°C for 1min, 20 cycles of 98°C for 10s, 65°C for 20s, 72°C for 30 seconds. Final elongation was done at 72°C for 2 min. Amplified libraries were purified twice using equal volumes of AMPure^®^ XP beads. Quality of the libraries as well as size of the amplicon and concentrations were determined using Agilent 2100 Bioanalyzer (Agilent Technologies, Diegem, BE) with the High Sensitivity DNA Kit (Agilent Technologies). 10 libraries were pooled equimolar on an Ion 316^™^ Chip (Thermo Fisher Scientific) and sequenced on a PGM sequencer, with all quality trimming options disabled on the Torrent Suite^™^ v4.0.2.

### Data processing pipeline for the HTS datasets

Untrimmed raw reads were demultiplexed by their MIDs, retaining only sequences containing the full UID primer sequence for further analysis, with no mismatches allowed. The UID sequence was extracted and categorized in relation to the starting position of the detected primer including the GATC spacer and stored in the sequence identifier. After clipping the MID, UID and constant region primer, the trimmed reads were quality controlled (80% of the bases Phred-like quality score above 20) and grouped into UID families. Using pagan-msa [47], a consensus sequence was generated for each UID-family containing more than 2 members. Afterwards, reverse primers were identified with up to 2 mismatches and clipped. Subsequently, sequences were collapsed to unique reads, storing counts in the read identifier, and uploaded to IMGT HighV-QUEST for error detection, correction, annotation and translation into amino acids. Post-IMGT HighV-QUEST datasets were separated into four categories (“productive”, “productive with detected errors”, “unproductive” and “unknown/else”) and processed separately. Data processing was performed using custom-made Python scripts (Python v2.7) employed in a parallelizing bash wrapper script using gnu-parallel [48] and the Biopieces framework (http://www.biopieces.org/).

### Graphs and statistics

All graphs and statistical analyses were performed using R base packages or GraphPad Prism 6. Average numbers are reported as mean ± standard deviation (SD) unless specified otherwise.

## SUPPLEMENTARY MATERIALS FIGURES AND TABLES





## Abbreviations

BM: Bone marrow
CDR3: complementary determining region 3
HTS: high-throughput sequencing
IG: immunoglobulin
IGH: immunoglobulin heavy chain
IMGT: ImMunoGeneTics
indel: insertions and deletions of nucleotides
MID: multiplex identifier
nt: nucleotide
PGM: (Ion Torrent) Personal Genome Machine
UID: Unique (molecular) identifier
ssUID: single side unique molecular identifier
