# Supplementary material for: High-throughput sequencing of murine immunoglobulin heavy chain repertoires using single side unique molecular identifiers on an Ion Torrent PGM
Source: Oncotarget. 2018 Jul 13;9(54):30225–39. doi: 10.18632/oncotarget.25493 (PMC6084394; doi:10.18632/oncotarget.25493)
Supplement: Supplementary file 1 [file oncotarget-09-30225-s001.pdf]

High-throughput sequencing of murine immunoglobulin heavy chain repertoires using single side unique molecular identifiers on an Ion Torrent PGM

SUPPLEMENTARY MATERIALS

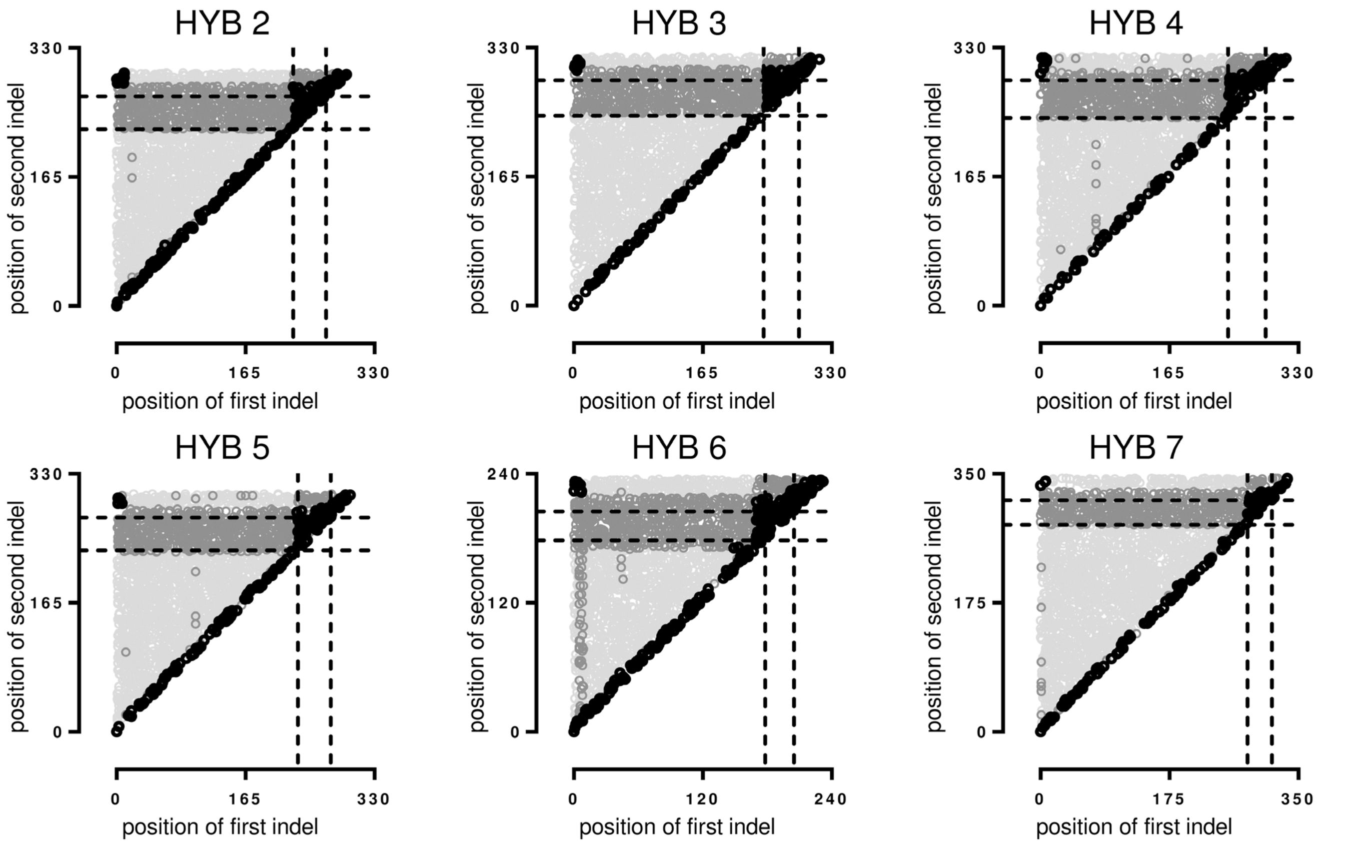

Supplementary Figure 1: Indel positions for mixed i1d1 datasets of hybridomas 2-7. The distances between the first and second indel before correction in the i1d1 dataset of hybridomas 2-7 are shown as scatterplots. Dotted lines indicate positions of IMGT IGH junctions. Productive sequences with detected indels are shown in grey, unproductive sequences are shown in dark grey. Sequences without detected errors are shown in black.

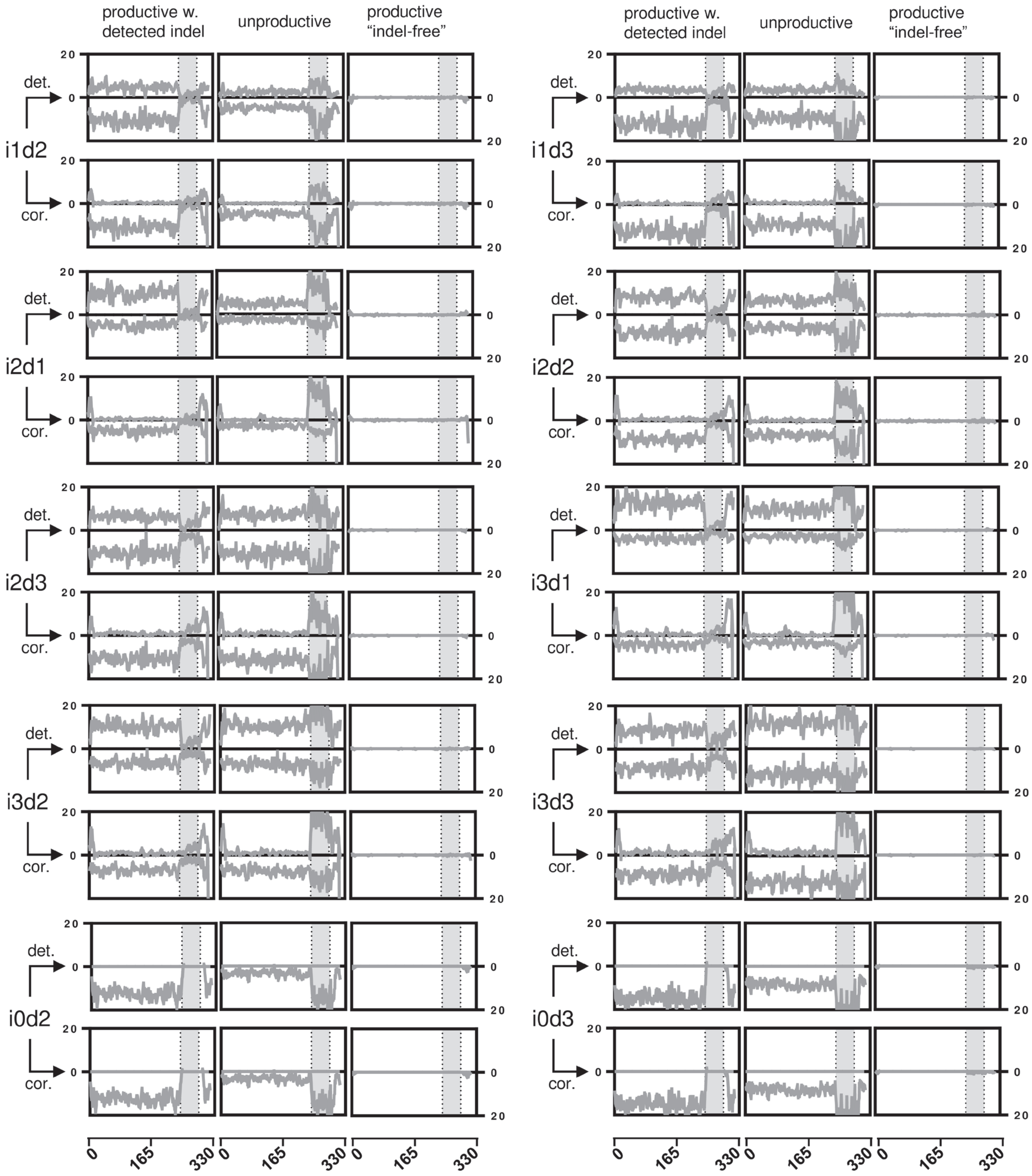

Supplementary Figure 2: Additional artificial indel set alignments. Indel positions are shown before and after IMGT error correction for artificially falsified Hybridoma 1 sequences separated by productivity. The indels for the datasets i1d2, i1d3, i2d1, i2d2, i2d3, i3d1, i3d2, i3d3, i0d2, i0d2 are shown per nucleotide position as line plot (smoothened over 4 neighbors). The grey area marks the IGH VDJ junction.

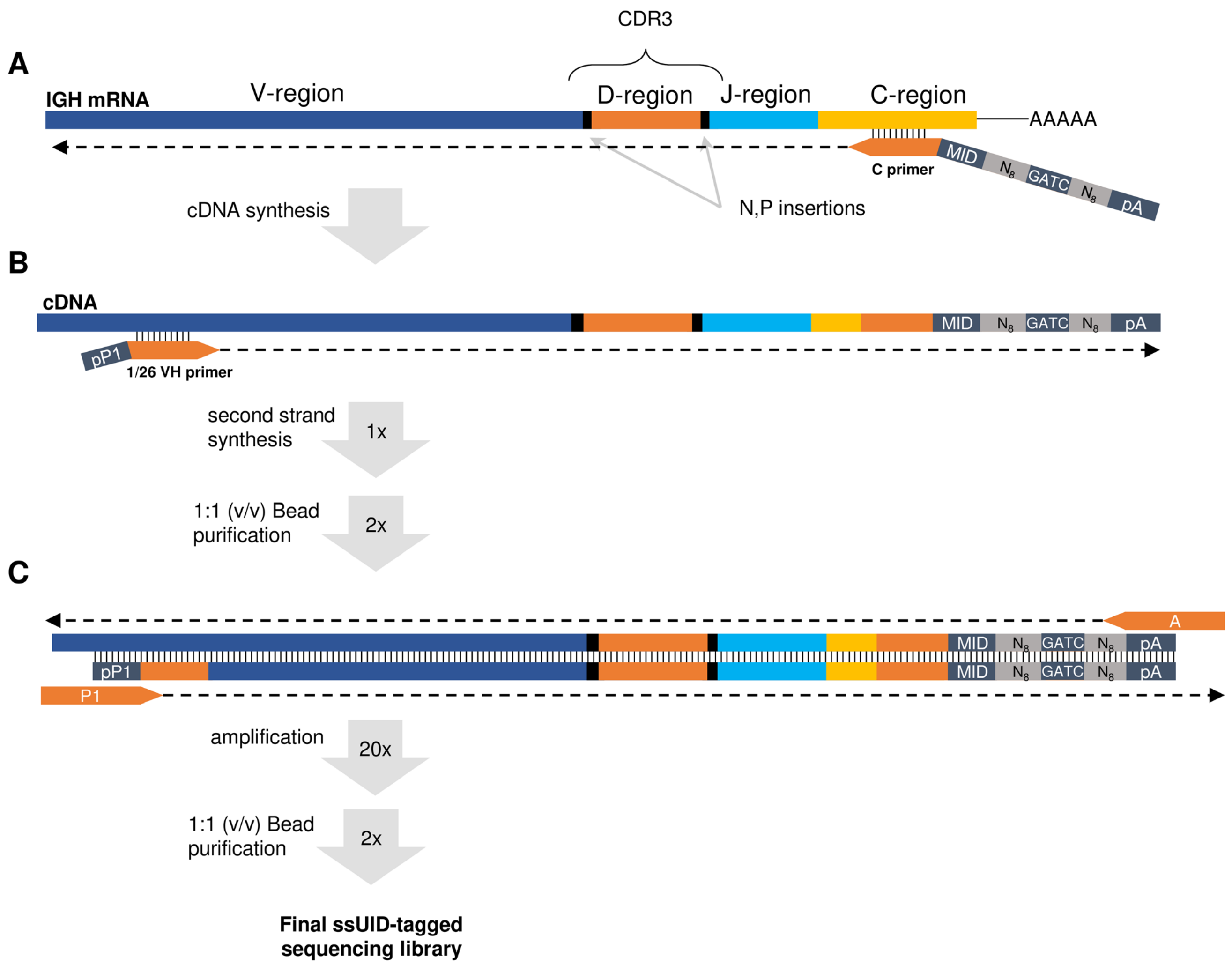

Supplementary Figure 3: 3-step PGM ssUID sequencing library preparation. A. In a first step, purified mRNA is used in a Superscript III reverse transcription. The Primer for the reverse transcription is specific for the murine IG C region and elongated by an MID for sample multiplexing as well as a UID consisting of 2x 8 random nucleotides (N8) separated by a 4-nucleotide spacer ('GATC'). The primer ends with the partial PGM sequencing adapter pA. **B.** In the second step, a mix of 26 IG VH region targeting primers (elongated by the partial PGM sequencing adapter pP1) is used in a single cycle PCR reaction to avoid amplification. The product of this reaction is purified twice with Agencourt® AMPureXP beads to remove the VH primers from the reaction mixture. **C.** In the final step, the purified reaction mixture is amplified using the full-length P1 and A adapters as primers in a 20 cycle PCR reaction. The product is as well purified twice to obtain the ssUID-tagged sequencing library.

Supplementary Table 1: IMGT V-QUEST analysis of Hybridoma sequences. Closest strain-native germline gene and mutation as determined by IMGT V-QUEST

Set	CDR3 Sequence	VH gene & allele	% germline
**HYB1**	SRWDYRYVYYPLDY	VH5S12*01	95.45%
**HYB2**	ARTYYGSYGFDY	VH9-1*03	95.61%
**HYB3**	ARQWLILWLGFAY	VH5-6-4*02	95.92%
**HYB4**	ARWDYRYVYYPLDY	VH5S12*01	96.69%
**HYB5**	TRGYYRYDGGFY	VH1S136*01	96.15%
**HYB6**	APKGLAY	VH1S40*01	90.56%
**HYB7**	ASRTTATGY	VH1S40*01	96.82%

Supplementary Table 2: Pre- and post-IMGT counts of the artificially falsified datasets of hybridomas 1-7.

For Supplementary Table 2 see attached word file in Supplementary Files.

Supplementary Table 3: Primer sequences and example layout for ssUID IonTorrent PGM HTS of murine IGH repertoires (Cγ & Cμ)

**Primer**	**Sequence**
IGHV1a	AGRTYCAGCTGCARCAGTCT
IGHV1b	AGGTCCAACTGCAGCAGCC
IGHV1c	TCAGTGAAGATGTCCTGCAAG
IGHV1d	AACTGGGTGAAGCAGAGGCCT
IGHV1e	AAGTTGTCCTGCACAGCTTCT
IGHV1f	AAGCTCAGCTGCAAGGCTTCT
IGHV2a	CCTCACAGAGCCTGTCCA
IGHV2b	CAGCCATCACAGACTCTGTCTC
IGHV3	GTGCAGCTTCAGGAGTCAG
IGHV4	GGAGGTGGCCTGGTGCAG
IGHV5a	AGCCTGGAGGGTCCCTGAA
IGHV5b	GCTTAGTGCAGCCTGGA
IGHV6	GAGGAGTCTGGAGGAGGCTT
IGHV7	TCTGGAGGAGGCTTGGTACA
IGHV8	CTGGGATATTGCAGCCCTCC
IGHV9	CAGTCTGGACCTGAGCTGAAG
IGHV10	GTGAGGTGCAGCTTGTTGAG
IGHV11	GAAGTGCAGCTGTTGGAGAC
IGHV12a	CCTGGTGAAACCCTCACAG
IGHV12b	GCTGTCATCAAGCCATCACAG
IGHV13	AGGCTTGGTGAGGCCTGGA
IGHV14	GAGGTTCAGCTGCAGCAGT
IGHV15	CAGGTTCACCTACAACAGTCTG
IGHV16	GTGCAGCTGGTGGAATCT
IGHCγ	GGCCAGTGGATAGACHGATG
IGHCμ	AGACATTTGGGAAGGACTGAC
A	CCATCTCATCCCTGCGTGTCTCCGACTCAG
P1	CCTCTCTATGGGCAGTCGGTGAT
**Example Layout:**	
pP1-IGHV4	GCGTGTCTCCGACTCAGGGAGGTGGCCTGGTGCAG
pA-N_8-GATC-N_8-IGHCγ	CTATGGGCAGTCGGTGAT*NNNNNNNNGATCNNNNNNNN*AGACATTTGGGAAGGACTGAC
	bold= partial A/P1 adapter
	italic=16N ssUID
