# Supplementary material for: High-throughput sequencing of murine immunoglobulin heavy chain repertoires using single side unique molecular identifiers on an Ion Torrent PGM
Source: Oncotarget. 2018 Jul 13;9(54):30225–39. doi: 10.18632/oncotarget.25493 (PMC6084394; doi:10.18632/oncotarget.25493)
Supplement: Supplementary file 2 [file oncotarget-09-30225-s002.docx]

**Supplementary table 2. Pre- and post-IMGT counts of the artificially falsified datasets of hybridomas 1-7.**

| hybridoma | permutation | pre-IMGT | prod. w. det. Indels | unproductive | productive (false) | unkown/else | corr. nt seq | corr. aa. seq. | corr. CDR3 |
| --- | --- | --- | --- | --- | --- | --- | --- | --- | --- |
| HYB1 | i0d1 | 2500 | 1861 | 475 | 164 | 0 | 0 | 21 | 2025 |
| HYB1 | i0d2 | 2500 | 1575 | 890 | 16 | 19 | 0 | 1 | 1591 |
| HYB1 | i0d3 | 2500 | 1289 | 1144 | 14 | 53 | 0 | 0 | 1293 |
| HYB1 | i1d0 | 2500 | 1835 | 443 | 196 | 26 | 1743 | 1804 | 2031 |
| HYB1 | i1d1 | 2500 | 1543 | 734 | 209 | 14 | 21 | 48 | 1709 |
| HYB1 | i1d2 | 2500 | 1447 | 985 | 35 | 33 | 0 | 2 | 1387 |
| HYB1 | i1d3 | 2500 | 1185 | 1248 | 8 | 59 | 0 | 0 | 1070 |
| HYB1 | i2d0 | 2500 | 1683 | 751 | 20 | 46 | 1118 | 1212 | 1703 |
| HYB1 | i2d1 | 2500 | 1404 | 1019 | 31 | 46 | 26 | 39 | 1350 |
| HYB1 | i2d2 | 2500 | 1217 | 1202 | 26 | 55 | 0 | 1 | 1121 |
| HYB1 | i2d3 | 2500 | 1061 | 1347 | 6 | 86 | 0 | 0 | 906 |
| HYB1 | i3d0 | 2500 | 1310 | 1089 | 17 | 84 | 708 | 800 | 1315 |
| HYB1 | i3d1 | 2500 | 1227 | 1184 | 7 | 82 | 22 | 33 | 1130 |
| HYB1 | i3d2 | 2500 | 1067 | 1326 | 8 | 99 | 0 | 3 | 921 |
| HYB1 | i3d3 | 2500 | 948 | 1426 | 2 | 124 | 0 | 0 | 725 |
| HYB2 | i0d1 | 2500 | 1792 | 461 | 247 | 0 | 0 | 16 | 2039 |
| HYB2 | i0d2 | 2500 | 1634 | 830 | 21 | 15 | 0 | 0 | 1655 |
| HYB2 | i0d3 | 2500 | 1329 | 1127 | 14 | 30 | 0 | 0 | 1331 |
| HYB2 | i1d0 | 2500 | 1798 | 480 | 222 | 0 | 1680 | 1693 | 2020 |
| HYB2 | i1d1 | 2500 | 1497 | 769 | 234 | 0 | 15 | 35 | 1678 |
| HYB2 | i1d2 | 2500 | 1374 | 1075 | 36 | 15 | 0 | 0 | 1320 |
| HYB2 | i1d3 | 2500 | 1234 | 1232 | 4 | 30 | 0 | 0 | 1141 |
| HYB2 | i2d0 | 2500 | 1669 | 803 | 23 | 5 | 1156 | 1176 | 1692 |
| HYB2 | i2d1 | 2500 | 1434 | 1033 | 28 | 5 | 38 | 60 | 1389 |
| HYB2 | i2d2 | 2500 | 1235 | 1217 | 33 | 15 | 3 | 4 | 1131 |
| HYB2 | i2d3 | 2500 | 1096 | 1355 | 15 | 34 | 0 | 0 | 933 |
| HYB2 | i3d0 | 2500 | 1342 | 1132 | 14 | 12 | 692 | 717 | 1348 |
| HYB2 | i3d1 | 2500 | 1250 | 1227 | 5 | 18 | 29 | 43 | 1168 |
| HYB2 | i3d2 | 2500 | 1077 | 1378 | 5 | 40 | 0 | 0 | 910 |
| HYB2 | i3d3 | 2500 | 978 | 1468 | 10 | 44 | 0 | 0 | 771 |
| HYB3 | i0d1 | 2500 | 1885 | 413 | 202 | 0 | 0 | 35 | 2083 |
| HYB3 | i0d2 | 2500 | 1738 | 741 | 19 | 2 | 0 | 0 | 1746 |
| HYB3 | i0d3 | 2500 | 1388 | 1090 | 12 | 10 | 0 | 0 | 1375 |
| HYB3 | i1d0 | 2500 | 1878 | 421 | 201 | 0 | 1855 | 1912 | 2079 |
| HYB3 | i1d1 | 2500 | 1533 | 752 | 215 | 0 | 17 | 40 | 1675 |
| HYB3 | i1d2 | 2500 | 1485 | 978 | 34 | 3 | 0 | 0 | 1393 |
| HYB3 | i1d3 | 2500 | 1354 | 1127 | 1 | 18 | 0 | 0 | 1199 |
| HYB3 | i2d0 | 2500 | 1712 | 761 | 20 | 7 | 1310 | 1397 | 1732 |
| HYB3 | i2d1 | 2500 | 1534 | 933 | 26 | 7 | 28 | 50 | 1449 |
| HYB3 | i2d2 | 2500 | 1341 | 1104 | 45 | 10 | 1 | 3 | 1192 |
| HYB3 | i2d3 | 2500 | 1228 | 1238 | 6 | 28 | 0 | 0 | 1027 |
| HYB3 | i3d0 | 2500 | 1355 | 1099 | 15 | 31 | 786 | 888 | 1351 |
| HYB3 | i3d1 | 2500 | 1329 | 1145 | 5 | 21 | 24 | 46 | 1176 |
| HYB3 | i3d2 | 2500 | 1240 | 1231 | 5 | 24 | 2 | 2 | 1017 |
| HYB3 | i3d3 | 2500 | 1095 | 1348 | 11 | 46 | 0 | 0 | 850 |
| HYB4 | i0d1 | 2500 | 1786 | 522 | 192 | 0 | 0 | 26 | 1978 |
| HYB4 | i0d2 | 2500 | 1599 | 859 | 26 | 16 | 0 | 0 | 1625 |
| HYB4 | i0d3 | 2500 | 1354 | 1092 | 15 | 39 | 0 | 0 | 1356 |
| HYB4 | i1d0 | 2500 | 1825 | 387 | 263 | 25 | 1751 | 1835 | 2088 |
| HYB4 | i1d1 | 2500 | 1546 | 746 | 181 | 27 | 18 | 55 | 1670 |
| HYB4 | i1d2 | 2500 | 1421 | 1011 | 28 | 40 | 0 | 0 | 1365 |
| HYB4 | i1d3 | 2500 | 1187 | 1252 | 4 | 57 | 0 | 0 | 1082 |
| HYB4 | i2d0 | 2500 | 1640 | 755 | 30 | 75 | 1164 | 1261 | 1669 |
| HYB4 | i2d1 | 2500 | 1451 | 944 | 30 | 75 | 35 | 60 | 1385 |
| HYB4 | i2d2 | 2500 | 1226 | 1154 | 34 | 86 | 0 | 2 | 1148 |
| HYB4 | i2d3 | 2500 | 1073 | 1330 | 5 | 92 | 0 | 0 | 907 |
| HYB4 | i3d0 | 2500 | 1309 | 1047 | 8 | 136 | 739 | 831 | 1309 |
| HYB4 | i3d1 | 2500 | 1209 | 1161 | 10 | 120 | 23 | 54 | 1120 |
| HYB4 | i3d2 | 2500 | 1116 | 1253 | 5 | 126 | 0 | 0 | 964 |
| HYB4 | i3d3 | 2500 | 947 | 1419 | 8 | 126 | 0 | 0 | 779 |
| HYB5 | i0d1 | 2500 | 1827 | 440 | 147 | 86 | 0 | 18 | 1974 |
| HYB5 | i0d2 | 2500 | 1523 | 786 | 16 | 175 | 0 | 0 | 1539 |
| HYB5 | i0d3 | 2500 | 1162 | 1085 | 9 | 244 | 0 | 0 | 1163 |
| HYB5 | i1d0 | 2500 | 1837 | 443 | 193 | 27 | 1845 | 1861 | 2030 |
| HYB5 | i1d1 | 2500 | 1476 | 747 | 184 | 93 | 19 | 36 | 1616 |
| HYB5 | i1d2 | 2500 | 1312 | 981 | 25 | 182 | 0 | 0 | 1254 |
| HYB5 | i1d3 | 2500 | 1078 | 1117 | 6 | 299 | 0 | 0 | 994 |
| HYB5 | i2d0 | 2500 | 1590 | 831 | 17 | 62 | 1281 | 1312 | 1607 |
| HYB5 | i2d1 | 2500 | 1337 | 988 | 21 | 154 | 27 | 50 | 1267 |
| HYB5 | i2d2 | 2500 | 1158 | 1118 | 26 | 198 | 1 | 1 | 1036 |
| HYB5 | i2d3 | 2500 | 931 | 1258 | 5 | 306 | 0 | 0 | 777 |
| HYB5 | i3d0 | 2500 | 1212 | 1177 | 10 | 101 | 783 | 821 | 1213 |
| HYB5 | i3d1 | 2500 | 1102 | 1225 | 5 | 168 | 36 | 53 | 1009 |
| HYB5 | i3d2 | 2500 | 906 | 1301 | 3 | 290 | 1 | 1 | 756 |
| HYB5 | i3d3 | 2500 | 826 | 1356 | 4 | 314 | 0 | 0 | 633 |
| HYB6 | i0d1 | 2500 | 1683 | 538 | 254 | 25 | 0 | 42 | 1937 |
| HYB6 | i0d2 | 2500 | 1457 | 958 | 28 | 57 | 0 | 0 | 1485 |
| HYB6 | i0d3 | 2500 | 1127 | 1226 | 28 | 119 | 0 | 0 | 1133 |
| HYB6 | i1d0 | 2500 | 1719 | 583 | 198 | 0 | 1608 | 1700 | 1917 |
| HYB6 | i1d1 | 2500 | 1304 | 855 | 308 | 33 | 29 | 65 | 1534 |
| HYB6 | i1d2 | 2500 | 1262 | 1131 | 52 | 55 | 0 | 3 | 1194 |
| HYB6 | i1d3 | 2500 | 1106 | 1285 | 7 | 102 | 0 | 0 | 991 |
| HYB6 | i2d0 | 2500 | 1426 | 1024 | 27 | 23 | 947 | 1049 | 1453 |
| HYB6 | i2d1 | 2500 | 1296 | 1123 | 40 | 41 | 31 | 60 | 1210 |
| HYB6 | i2d2 | 2500 | 1083 | 1279 | 74 | 64 | 1 | 5 | 1002 |
| HYB6 | i2d3 | 2500 | 946 | 1430 | 15 | 109 | 0 | 0 | 787 |
| HYB6 | i3d0 | 2500 | 1027 | 1370 | 38 | 65 | 469 | 547 | 1035 |
| HYB6 | i3d1 | 2500 | 1071 | 1339 | 11 | 79 | 35 | 54 | 962 |
| HYB6 | i3d2 | 2500 | 936 | 1438 | 15 | 111 | 0 | 4 | 777 |
| HYB6 | i3d3 | 2500 | 848 | 1495 | 21 | 136 | 0 | 0 | 628 |
| HYB7 | i0d1 | 2500 | 2001 | 333 | 114 | 52 | 0 | 18 | 2115 |
| HYB7 | i0d2 | 2500 | 1762 | 632 | 8 | 98 | 0 | 0 | 1770 |
| HYB7 | i0d3 | 2500 | 1455 | 858 | 4 | 183 | 0 | 0 | 1452 |
| HYB7 | i1d0 | 2500 | 1986 | 338 | 122 | 54 | 1921 | 1962 | 2108 |
| HYB7 | i1d1 | 2500 | 1649 | 574 | 158 | 119 | 26 | 44 | 1778 |
| HYB7 | i1d2 | 2500 | 1562 | 773 | 16 | 149 | 0 | 0 | 1525 |
| HYB7 | i1d3 | 2500 | 1289 | 982 | 2 | 227 | 0 | 0 | 1234 |
| HYB7 | i2d0 | 2500 | 1797 | 567 | 9 | 127 | 1402 | 1452 | 1806 |
| HYB7 | i2d1 | 2500 | 1541 | 794 | 9 | 156 | 31 | 61 | 1506 |
| HYB7 | i2d2 | 2500 | 1352 | 942 | 16 | 190 | 0 | 0 | 1296 |
| HYB7 | i2d3 | 2500 | 1160 | 1082 | 3 | 255 | 0 | 0 | 1073 |
| HYB7 | i3d0 | 2500 | 1500 | 815 | 4 | 181 | 990 | 1054 | 1501 |
| HYB7 | i3d1 | 2500 | 1326 | 971 | 2 | 201 | 36 | 61 | 1271 |
| HYB7 | i3d2 | 2500 | 1216 | 1027 | 3 | 254 | 1 | 1 | 1094 |
| HYB7 | i3d3 | 2500 | 1102 | 1109 | 4 | 285 | 0 | 0 | 959 |
